# Determination of Anlotinib, a Tyrosine Kinase Inhibitor, in Rat Plasma by UHPLC-MS/MS and Its Application to a Pharmacokinetic Study

**DOI:** 10.1155/2019/5016757

**Published:** 2019-12-10

**Authors:** Zhe Wang, Le-jing Lian, Yan-yan Dong, Xiao Cui, Jian-chang Qian, Cheng-ke Huang, Rui-jie Chen, Wei Sun

**Affiliations:** ^1^Department of Pharmacy, The Second Affiliated Hospital and Yuying Children's Hospital of Wenzhou Medical University, Wenzhou, Zhejiang 325027, China; ^2^Department of Ultrasonography, The Second Affiliated Hospital of Wenzhou Medical University, Wenzhou, Zhejiang 325027, China; ^3^School of Pharmaceutical Sciences, Wenzhou Medical University, Wenzhou, Zhejiang 325035, China

## Abstract

Anlotinib is a novel inhibitor of receptor kinase tyrosine with multitargets and has a broad spectrum of inhibitory action on tumor angiogenesis and growth. A simple and rapid UHPLC-MS/MS bioanalytical method was validated for the determination of anlotinib in rat plasma, using imatinib as an internal standard. An Acquity BEH C18 column was used to separate analytes. The eluents consisted of formic acid/water (0.1 : 100, v/v) and acetonitrile with a mobile phase. A triple quadrupole mass spectrometer was operated for the quantification with multiple reaction monitoring (MRM) to determine transitions: 408.2 ⟶ 339.1 for anlotinib, and 494.3 ⟶ 394.1 for imatinib. The validated range was 0.1–50 ng/mL for anlotinib. Mean recovery rate of anlotinib in plasma was ≥99.32% and reproducible. Also, the intra- and interday precisions were both below 15%. This robust method was successfully applied to support the pharmacokinetic study of anlotinib in rats.

## 1. Introduction

A tyrosine kinase (TK) is an enzyme that catalyzes the transfer of phosphate from ATP to tyrosine residues in a cell. It is also crucial for regulation of cell proliferation, differentiation, function, survival, and motility. Currently, TKs are regarded as ideal targets for cancer treatment because of their dysregulation in cancer cell [1, 2]. Multitargeted tyrosine kinase inhibitors (TKIs) have demonstrated significant anticancer effects in a wide variety of tumor types via inhibiting angiogenetic and proliferative signaling [2, 3].

Anlotinib, a novel oral multitargeted TKI, inhibits tumor angiogenesis and proliferation with high potency and broad specificity [4]. *In vitro*, studies indicated that anlotinib suppressed tumor cell growth through the inhibition of platelet-derived growth factor receptor *β* (PDGFR *β*), vascular endothelial growth factor receptor 2/3 (VEGFR2/3), and the stem cell-factor receptor (c-Kit) [5–8]. *In vivo*, anlotinib has showed broad activity against human tumor xenograft models, especially colon, ovarian, liver, renal, glioma, and non-small-cell lung [9]. Besides possessing preclinical *in vitro* and *in vivo* antitumor activity, anlotinib was also undergoing phase 2 and 3 clinical trial for the treatment of various refractory solid tumor and carcinomas. A multicentre, randomized phase II clinical trial (ALTER0302) showed that comparing with the placebo, anlotinib provided obvious progression-free survival (PFS) benefits for patients who suffer refractory advanced non-small-cell lung cancer and showed good toxicity tolerance [10]. In addition, Han et al. conducted a multicentre, double-blind, and randomized phase 3 clinical study to confirm the function of anlotinib in advanced non-small-cell lung cancer sufferers who have undergone progression or recurrence after second-line or further treatment [11]. The results indicated that anlotinib appear to improve overall survival (OS) and progression-free survival (PFS), thus anlotinib could be regarded as a potential third-line or further treatment in patients with advanced non-small-cell lung cancer [11].

However, to date, there is no bioanalytical report of the systematic validation for quantification of anlotinib in a biological matrix. There were only two analytical methods reported for the determination of anlotinib in biological fluids [12, 13]. The method Zhong et al. established had some drawbacks, such as requiring large volumes of plasma samples (250 μl) and low sensitivity (0.2 ng/ml). Du et al. chose diazepam as internal standard for anlotinib; however, diazepam did not have the similar chromatographic and extraction behaviors to anlotinib. Otherwise, they established a method for the simultaneous quantitative determination of anlotinib, ceritinib, and ibrutinib in rat plasma; however, in actual clinical diagnosis and treatment, those three drugs may not be used by patients simultaneously. In addition, the total analysis time of this method was 5 min which was longer than our method (3.5 min). In the current study, we described a simple and selective UHPLC-MS/MS assay for simultaneous quantitation of anlotinib using imatinib as internal standard (IS) in rat plasma. This method was successfully applied to the quantitative analysis of anlotinib for rat pharmacokinetic study.

## 2. Materials and Methods

### 2.1. Chemicals and Materials

Anlotinib (purity > 98%) was gifted from Jiangsu Chia-Tai Tianqing Pharmaceutical Co., Ltd. Imatinib (internal standard, IS; purity > 98%) was purchased from Sigma-Aldrich (St. Louis, USA). Methanol (purity > 99.9%) and formic acid (purity > 98%) for LC-MS-grade were from EMD Millipore Corporation. LC/MS-grade acetonitrile was purchased from Merck (Darmstadt, Germany). Other reagents of analytical grade in the present study were applied without further purification. Water (LC grade) was produced by the Laboratory Water Purification System (Pall Co., Shanghai, China).

### 2.2. UHPLC-MS/MS Conditions

An Agilent UHPLC unit system (MA, USA) was equipped with a degasser and QuatPump along with an autosampler. The aliquots of all samples were injected into the UHPLC system for analysis, and the injection volume was 5 *μ*L. Chromatographic separation of analyte and IS were conducted with an Acquity BEH C18 column (2.1 × 50 mm, 1.8 *μ*m; Agilent Technologies, USA). The column temperature was set at 30°C. The eluents consisted of formic acid/water (0.1 : 100, v/v) as mobile phase A and acetonitrile as mobile phase B. The flow rate was maintained at 0.4 mL/min with 20% mobile phase B to 95% mobile B, from 0 to 0.4 min, followed by holding 95% mobile phase B from 0.4 to 1.5 min. Also, the gradient was back to the initial, which maintained for 2.0 min to re-equilibrate. The needle wash solvent was comprised of methanol and water (50 : 50, v/v). The whole run time of this assay barely took 3.5 min.

### 2.3. Mass Spectrometry

An Agilent 6420 triple quadrupole mass spectrometer (Agilent Corporation, MA, USA) was coupled to the LC system. The mass spectrometer adapted to the positive electrospray ionization source (ESI) mode with the following parameters: the desolvation gas (nitrogen) flow rate was set at 800 L/h; the gas temperature was 350°C; and the capillary voltage was set at 4.00 kV. A multiple reaction monitoring (MRM) mode was chosen to detect and determine anlotinib and IS, using high-purity nitrogen as the collision gas at a pressure of about 0.1 MPa. The precursor-to-product ion transitions subjected to the MRM detection were m/z 408.2 ⟶ 339.1 for anlotinib and m/z 494.3 ⟶ 394.1 for imatinib. Fragmentor was set at 110 V for anlotinib and at 112 V for imatinib. Collision Energy was set at 17 eV.

### 2.4. Preparation of the Stock Solutions and Working Solutions

The primitive stock solutions for anlotinib and IS were diluted in methanol. Working solutions of anlotinib and IS (2.0 *μ*g/mL) were prepared by diluting the stock solutions in methanol. Also, these working solutions were stored at 4°C.

### 2.5. Calibration Curve and Quality Control Samples

Calibration standards (0.1, 0.25, 0.5, 1, 2.5, 5, 10, 20, and 50 ng/mL) and quality control samples (QCs) at three level concentrations of 0.25, 5, and 40 ng/mL were prepared by spiking blank rat plasma with appropriate working standards of drug and IS.

### 2.6. Sample Preparation

To precipitate protein, an aliquot of 2.5 *μ*L IS solution and 150 *μ*L acetonitrile were added to 50 *μ*L plasma samples. These samples were followed by vortexing for 1 min and centrifuged for 10 minutes at 16,000*g* at 4°C. The supernatant was transferred into injection vials, which were loaded into autosampler, and 5 *μ*L aliquots of these supernatants were injected into the LC-MS/MS system for analysis.

### 2.7. Method Validation

Method validation was based on the European Medicines Agency (EMA) Guideline on bioanalytical method validation 2012 [14] and US FDA Guidance for Industry on Bioanalytical Method Validation [15]. The method in the present study was validated for specificity, precision, accuracy, matrix effect, stability, and recovery according to the latest report [16].

#### 2.7.1. Specificity

The method's specificity was determined by analyzing the chromatograms of six blank plasma from different rats and spiked plasma. Blank plasma sample, blank plasma sample spiked with anlotinib, and plasma sample at 10 min after oral administration of anlotinib were analyzed, and it is not regarded as interfering peak that the peak area is unexceeding 20% of peak area of the lower limit of quantitation (LLOQ) level.

#### 2.7.2. Calibration Curve

The method's linearity was evaluated based on three independent calibration curves established by plotting the peak area ratios of anlotinib to the IS (*Y*) versus the known anlotinib concentration (*X*) with a weighted (1/*x*^2^) least squares linear regression model. The correlation coefficients (*R*^2^) greater than 0.99 were the prerequisite for determination of linearity of the analyte. The LLOQ was defined as the lowest concentration point on the calibration curve. Carry-over effects were defined when the signal to noise ratio (S/N) was more than 20% of that of LLOQ. Carry-over effects were determined by detecting extracted blank rat plasma directly after extracted samples at the upper limit of quantification (ULOQ).

#### 2.7.3. Accuracy and Precision

Intraday and interday accuracy and precision were evaluated by analyzing QCs at three differrent levels (0.25, 5, and 40 ng/mL) in six replicates. Intraday accuracy and precision were determined during one day. Interday accuracy and precision were determined on three consecutive days. The precision was expressed in terms of percent relative standard deviation (RSD, %). The accuracy was expressed in terms of percent relative error (RE, %) between nominal and measured parameters. The acceptable accuracy (relative error, RE) for QCs ought to be within ±15%, and precision (relative standard deviation, RSD) ought to be within 15%.

#### 2.7.4. Recovery and Matrix Effects

The method's recovery was evaluated at three concentrations (0.25, 5, and 40 ng/mL) of QC samples by comparing the peak areas of plasma samples with the neat solution spiked into blank plasma before and after extraction.

The method's matrix effects were assessed by comparing the response of anlotinib spiked into blank plasma extraction at three concentrations (0.25, 5, and 40 ng/mL) with those of standard samples in the same concentration.

#### 2.7.5. Stability Experiments

The stability of three concentrations (0.25, 5, 40 ng/mL) of QCs was analyzed under typical storage conditions: (1) rat plasma stored at room temperature for 12 hours (short-term stability); (2) rat plasma stored at −80°C for 30 days (long-term stability); and (3) under three freeze-thaw cycles from −80°C to room temperature. The stability of stock solution of anlotinib and IS was analyzed under room temperature for 24 hours as well as at 4°C for 2 weeks.

#### 2.7.6. Incurred Samples Reanalysis (ISR)

According to the EMA and FDA guidelines, the incurred samples reanalysis (ISR) helps to assure bioanalysis reliability [17]. The guidelines recommend 10% of the samples should be reanalyzed for this test when the number of samples is less than 1000 and be around Cmax and in the elimination phase. The obtained difference between the initial value and the ISR ought to be within ±20% for reanalysis values of two-thirds of all samples.

### 2.8. Pharmacokinetic Study

Male Sprague Dawley rats weighing 250 ± 20 g were purchased from the Shanghai Laboratory Animal Co. (Shanghai, China). All experimental rats were housed in a standard cage with a humidity of 50 ± 20%, a constant temperature of 23–25°C, and a light/dark cycle condition for at least 7 days before the pharmacokinetic study. After 8 h fasting (animals had free access to water), the rats received a single oral administration with anlotinib at a dosage of 10 mg/kg. All animal experiments in present study were approved by the Institutional Animal Care and Use Committee of Wenzhou Medical University (Wenzhou, China).

Blood samples (150 *μ*L) were collected from the tail vein before the dose and at 0.17, 0.75, 1, 1.5, 2, 3, 4, 5, 7, 9, 12, 24, and 48 h after the oral administration of anlotinib. These series of blood samples were transferred into 1.5 mL heparinized tubes. The rat blood samples were immediately centrifuged for 10 min at 5,000 rpm at 4°C, and then the separation of plasma was stored at −80°C until analysis. The noncompartmental statistical model was performed to calculate the pharmacokinetic parameters of anlotinib by Drug and Statistics 3.0 software.

## 3. Results

### 3.1. Mass Spectroscopy and Liquid Chromatography

**Figure 1:**
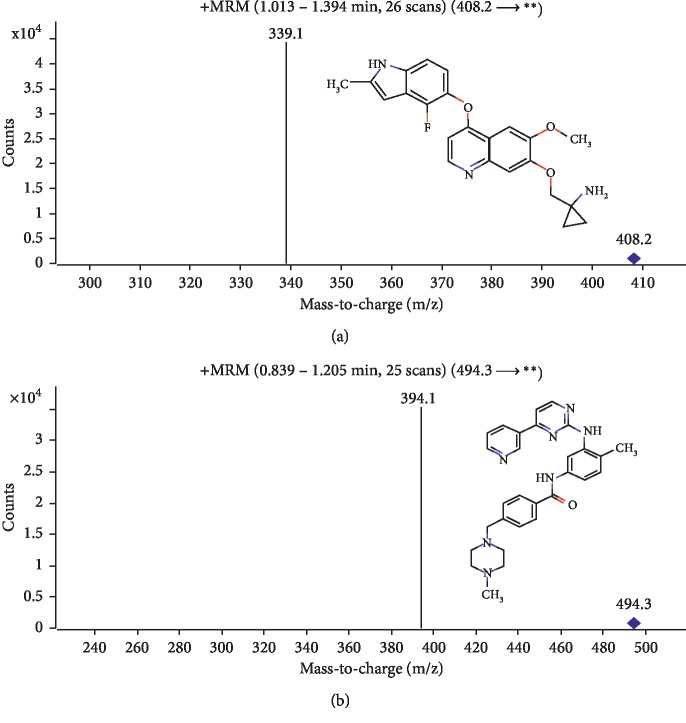
Product ion mass spectra of anlotinib ([M+H]^+^ m/z 408.2 ⟶ 339.1) and imatinib ([M+H]^+^ m/z 494.3 ⟶ 394.1) in the positive ionization mode.

Comparing with the ESI negative mode, the ESI positive ion mode showed higher sensitivity for anlotinib. The program Optimizer (Version B. 07.01 MassHunter Workstation Software) was applied to optimize the precursor and productions and give the highest response and ideal detection parameter. Finally, MRM of the transitions at m/z 408.2 ⟶ 339.1 and m/z 494.3 ⟶ 394.1 were chosen for anlotinib and IS, respectively (Figure 1). Also, the mass spectrometer parameters, including fragmentor and collision energy, are summarized in Table 1. To optimize the signal response and the peak shape of analytes, various compositions of the mobile phase were evaluated. Finally, the best choices of the ingredient of the mobile phase in this study were acetonitrile and 0.1% formic acid.

### 3.2. Internal Standard

The structure of IS ought to be comparable to the analyte such that it behaves similarly during the sample preparation and analysis [18]. In this study, we chose imatinib as the IS for the analysis. Imatinib is a selective inhibitor of the protein tyrosine kinase [19]. The retention time and recovery of imatinib were similar with those of anlotinib in the separation procedure and in the preparation process.

### 3.3. Optimization of Sample Preparation

Liquid-liquid extraction that adopts ethyl acetate extraction for quantification of anlotinib in biological matrices was reported in a previous study [12]. However, a major drawback of the liquid-liquid extraction method was time consumption. The method of protein precipitation with methanol or acetonitrile was also applied for sample preparation to quantify the analytes in plasma for its simple operation. In this study, the method of protein precipitation with acetonitrile promoted extraction rate and showed a better peak shape for anlotinib, when compared with methanol. Thus, acetonitrile was chosen for the protein precipitation to extract anlotinib and IS in the sample preparation.

### 3.4. Specificity and Selectivity

**Figure 2:**
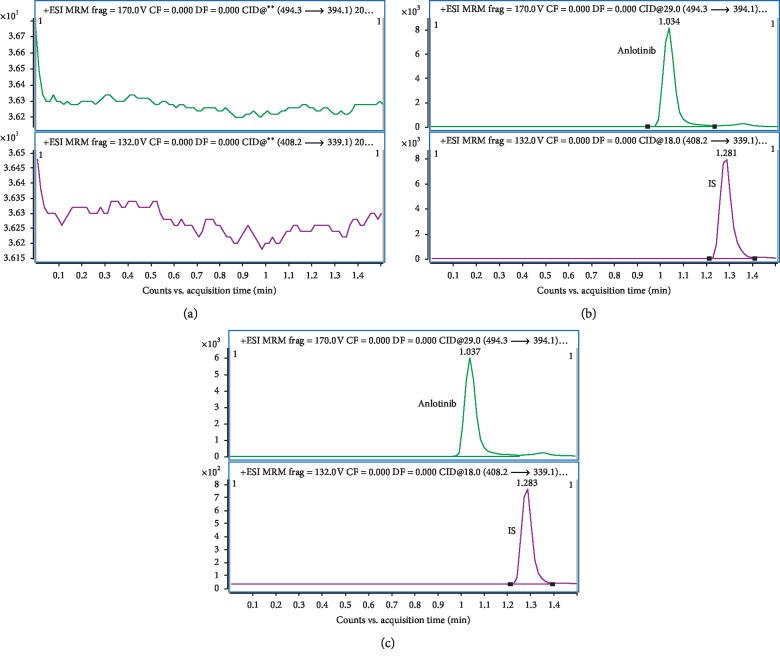
Representative MRM chromatograms of anlotinib and imatinib (IS) in rat plasma. (a) Blank samples from plasma (A); (b) sample at 40 ng/mL for anlotinib in plasma; (c) sample at 10 min after oral administration of 10 mg/kg anlotinib to rat.

Figure 2 shows representative UHPLC-MS/MS chromatograms of blank plasma (A), blank plasma spiked anlotinib and IS (B), and plasma sample taken 10 min after oral treatment with anlotinib at a dosage of 10 mg/kg (C). No interfering peak from blank plasma was detected at the retention times of anlotinib and IS. Moreover, no crosstalk was observed in anlotinib and IS.

### 3.5. Calibration Curve

Calibration curves were analyzed at eight levels concentration, in the range from 0.10 to 50 ng/mL. The typical standard curve was *y* = 7.5549*x* + 0.0267 (*r*^2^ = 0.9998) for anlotinib. The LLOQ was 0.1 ng/mL for anlotinib, which was sensitive enough for further pharmacokinetic study.

### 3.6. Accuracy and Precision

The accuracy and precision were evaluated for intraday and interday of anlotinib at LLOQ and the three QCs (0.25, 5, 40 ng/mL). The results are shown in Table 2. The mean of RE for intraday and interday accuracies was within the range of 85.0–115%, and precision (RSD, %) was below 15%. These results indicated that the approach in the present study was precise and accurate.

### 3.7. Recovery and Matrix Effect

The recoveries and matrix effects of anlotinib and IS were investigated and assembled in Table 3. Recoveries at QC concentrations (0.25, 5, and 40 ng/mL) were 101.79 ± 9.41, 102.22 ± 9.40, and 105.48 ± 6.64%, respectively. The recovery of the IS (2.0 *μ*g/mL) was 106.09 ± 7.65%. Matrix effects at concentrations of 0.25, 5, and 40 ng/mL were 104.89 ± 4.46%, 111.67 ± 3.68%, and 102.04 ± 5.97%, respectively. The matrix effect of IS (2.0 *μ*g/mL) was 101.66 ± 4.53%. The results demonstrated that no obvious matrix effects were observed for anlotinib and IS.

### 3.8. Stability

The results of the stability in different conditions are presented in Table 4. Stabilities of the anlotinib stock solution was 95.96% for the long-term test and 98.96% for the short-term test. The stability of IS stock solution was 98.73% for the long-term experiment and 99.35% for short-term experiment. These values showed that stock solutions were stabilizing in different storage conditions.

### 3.9. Incurred Samples Reanalysis

Ten samples in the acceptance criteria were applied for ISR. The accuracy value range was between 88.47% and 108.95% for the initial assay results.

### 3.10. Pharmacokinetic Study

**Figure 3:**
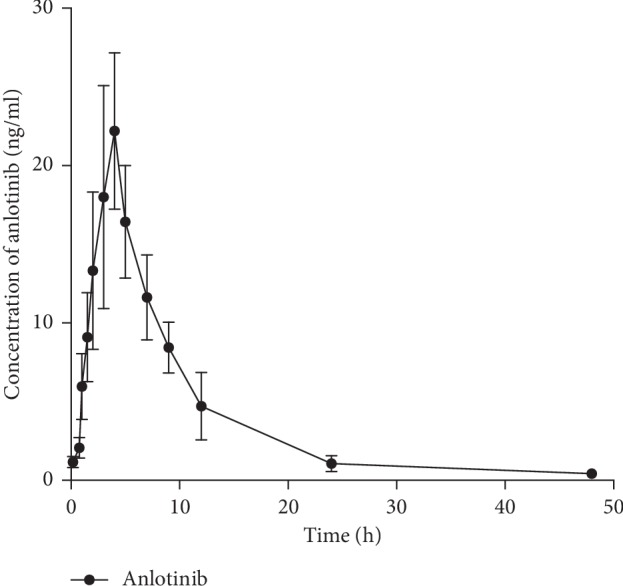
The pharmacokinetic profiles of anlotinib after oral administration to rats (*n* = 6).

The developed method was successfully applied to determine the concentration of anlotinib in rat plasma samples collected in the pharmacokinetic study. Plasma concentration profile of anlotinib versus time from rats following anlotinib (10 mg/kg) oral administration was shown in Figure 3. As shown in Table 5, the main pharmacokinetic parameters were collected. In the present study, the value of *t*_1/2_ was 5.05 ± 0.62 h, which was similar with that in the reported literature (5.7 ± 3.1 h) [12]. The AUC_0-*t*_ was 184.43 ± 49.83 ng/mL·h, and *C*_max_ was 24.22 ± 4.31 ng/mL. The value of these PK parameters was different from that in the reported literature, which might due to the different doses and individual difference in the absorption.

## 4. Discussion

Anlotinib is a novel multitarget receptor tyrosine kinase inhibitor. In the reported study, an LC-MS method was also performed to quantify anlotinib in the biological matrix. In our method, 50 *μ*L of plasma for anlotinib was enough to assure a sufficient sensitivity.

Protein precipitation method by acetonitrile has been selected for the protein precipitation to assay other TKIs in the reported studies [20–22]. In this study, we used the protein precipitation method with acetonitrile in sample preparation for its simple isolation procedure. The accuracy, precision, recovery, and matrix effect were also evaluated and found to be in the acceptable range. The advantages of our method are as follows: a small volume of plasma required for the analyses, simple and rapid extraction procedures, and short analysis time.

After a single oral administration with anlotinib at a dosage of 10 mg/kg, maximum concentrations in plasma (*C*_max_: 24.22 ng/mL) attained at 3.33 hours (*T*_max_). Half-life (*t*_1/2_) was found to be 5.05 h, which is similar to the reported study [12]. The apparent volume of distribution (V/F) for anlotinib was 406.02 L/kg.

## 5. Conclusion

A novel, reliable, and precise method was developed for the quantification of anlotinib in rat plasma. The LLOQ was sufficient for the pharmacokinetic study. The results of this validation study indicated that good accuracy and precision were fully validated based on guidelines and met the criteria acceptance. In addition, the method can be utilized as a useful approach for quick detection, which realizes the pharmacokinetic study in preclinical studies.

## Figures and Tables

**Table 1:** MS parameters for anlotinib and imatinib (IS).

Drugs	Precursor ion	Product ion	Fragmentor (V)	Collision energy (eV)
Anlotinib	408.2	339.1	132	18
Imatinib	494.3	394.1	170	29

**Table 2:** Intra- and interday precision and accuracy values for anlotinib in rat plasma (*n* = 3 days, 6 replicates per day).

Concentration (ng/mL)	Precision RSD (%)		Accuracy RE (%)	
	Intraday	Interday	Intraday	Interday
0.25	3.39	7.32	1.41	3.53
5	4.9	4.59	3.81	3.68
40	2.72	3.92	4.62	2.69
0.1	10.81	11.64	1.68	3.17

**Table 3:** Mean extraction recovery and matrix effects of anlotinib and the IS in rat plasma (*n* = 6).

Compound	Concentration (ng/mL)	Extraction recoveries (%)		Matrix effects (%)	
		Mean ± SD	RSD (%)	Mean ± SD	RSD (%)
Anlotinib	0.25	99.32 ± 6.74	6.79	104.27 ± 3.65	3.51
	5	102.27 ± 6.48	6.33	102.42 ± 4.17	4.07

IS	40	104.14 ± 3.95	3.8	101.33 ± 4.08	4.03
	100	106.09 ± 8.11	7.65	101.66 ± 4.53	4.45

**Table 4:** Stability of anlotinib in rat plasma under various storage conditions (*n* = 3).

		Short-term stability	Long-term stability	Freeze/thaw stability	Autosampler stability
0.25 ng/ml	Mean	0.26	0.25	0.26	0.24
	RSD	3.9	2.18	4.1	4.42
	RE	4.65	−1.55	2.03	−2.27

5 ng/ml	Mean	5.21	5.19	4.94	5.14
	RSD	3.99	3.32	3.47	4.32
	RE	4.27	3.75	−1.13	2.75

40 ng/ml	Mean	41.64	41.19	40.55	41.14
	RSD	3.26	3.97	3.91	4.81
	RE	4.1	2.97	1.39	2.85

**Table 5:** Pharmacokinetic parameters of anlotinib in plasma following an oral administration at a dose of 10 mg/kg in rats (*n* = 6).

Parameters	Mean ± SD
AUC_0-*t*_ (ng/mL·h)	184.431 ± 49.827
AUC_0-∞_ (ng/mL·h)	185.594 ± 49.234
MRT_(0-*t*)_ (h)	8.982 ± 1.452
MRT_(0-∞)_ (h)	9.199 ± 1.214
*t *_1/2_ * z* (h)	5.05 ± 0.62
*T *_max_ (h)	3.333 ± 0.816
*C *_max_ (ng/mL)	24.219 ± 4.311
CL/F (L/h/kg)	56.512 ± 12.228
V/F (L/kg)	406.029 ± 72.748

## Data Availability

The data used to support the findings of this study are available from the corresponding author upon request.
